# In Search of a Dentist

**Published:** 1900-11-15

**Authors:** L. P. Haskell


### IN SEARCH OF A DENTIST.

Having to remain for a day in Liverpool waiting for the steamer to leave, I thought to call upon some of the dentists. Starting out at 8 o'clock to look around the city I first went through many business streets, and saw but one sign, but the surroundings were such I did not venture in. Then I tramped through many residence or mixed residence and business streets and still without success. I went into a drug store and inquired. The proprietor said there was one near by, but I failed to find him. After walking a considerable distance and seeing no evidence of a dentist but a shop window filled with specimens and prices ranging from two shillings (25 cents) and upward per tooth, I again called upon a druggist in a good residence locality. He said he "pulled teeth." Told him I wanted to find a *dentist*. He said he knew of none anywhere within two miles. Went back to a business section where I had not been, and again called upon a druggist. He said on a certain street I would find several. I did so, found four offices, but the dentists had not yet come, as it was not time for them, and yet it was ten o'clock. At last I found one in. He came forward and I handed him my card. He looked at it and said: "Well what do you want?" I replied that I simply was making a friendly call. " Well, will you tell me just what you want of me ?" I replied, " Nothing, but had *expected* to meet a gentleman," and left.

This was my experience in a city of 800,000 population. I wondered whether here was not a good opening for some of the surplus dentists of America now so rapidly accumulating.—*L. P. Haskell, in Dental Review.*

